# Nuclear error phenotypes in the two-cell embryo are correlated to blastocyst formation rate after assisted reproduction

**DOI:** 10.1007/s10815-024-03354-9

**Published:** 2024-12-27

**Authors:** Amanda Stenberg, Juliane Baumgart, Emma Adolfsson

**Affiliations:** 1 Department of Obstetrics and Gynecology, Örebro University Hospital, Örebro, Sweden; 2 Department of Obstetrics and Gynecology, Faculty of Medicine and Health, Örebro University, Örebro, Sweden

**Keywords:** Nuclear error phenotypes, Assisted reproduction technology, Two-cell stage embryos, Time-lapse imaging, Blastocyst formation rate

## Abstract

**Purpose:**

Map the nuclear error phenotypes in the two-cell embryo after assisted reproduction using time lapse images and the effect on good quality blastocyst formation.

**Methods:**

Retrospective cohort study using time lapse images, categorizing 2331 two-cell embryos from 392 patient couples and 504 ART cycles categorizing each embryo as mononucleated, multinucleated, micronucleated, binucleated, split nucleation or mixed error. Correlating nuclear error phenotype with good quality blastocyst formation rate (BFR) using contingency tables and unadjusted odds ratio.

**Results:**

An overall nuclear error rate of 47.1% was observed in two-cell embryos. The most frequent error was multi-nucleation (14.2%) followed by mixed error (11%), micro-nucleation (8.6%), bi-nucleation (7.4%) and split nucleation (5.8%). Blastocyst formation rate (BFR) was reduced in embryos with nuclear errors, 46.2% for embryos with one cell affected, 27.6% for embryos with both cells affected, compared to 58.6% for mononucleated cells, *p* < 0.001 for both. Binucleated embryos were as likely as mononucleated embryos to become clinically useful blastocysts (56.8% vs 58.6%, n.s., unadjusted OR 0.94), whereas all the other phenotypes were less likely to develop into good quality blastocysts. The worst outcome was noted for embryos with split nucleation, with just 12.4% BFR, OR 0.12 (0–08-0.21), *p* < 0.001.

**Conclusion:**

Nuclear errors are common at the two-cell stage. Overall, presence of nuclear errors reduces the likelihood of becoming good quality blastocysts. Both the number of affected cells and the different nuclear error phenotypes have impact on blastocyst formation rate, except binucleated embryos.

## Introduction

Human reproduction is inefficient. Only one of three fertilized oocytes results in a live birth [1] with even lower numbers after assisted reproduction treatment (ART) [2–5]. In countries where PGT-A is prohibited non-invasive observations are the only tools available for embryo selection, and without genetic screening the implantation rate for a blastocyst seldom exceeds 50% [6].

The first mitosis resulting in the two cell embryo is highly error prone [7], and the first division affects both daughter cells. The first mitotic division happens at ~ 27 h post insemination; in ART this corresponds to night, and in the morning when the embryologists return to the lab, the embryo has completed the second mitosis and reached the four-cell stage. Assessing the two-cell stage therefore, in clinical practice, relies on having access to time lapse images. Time lapse imaging technologies (TLT) have provided the embryologists with the possibility of continuous monitoring of the developing embryos without interruption and allowed for detailed studies of the embryos normally missed by static observation, including the first mitosis and the two-cell stage. The nuclear status of the cells in human early embryo can infer important information about the embryo development potential and chance of successful outcome for the embryo. The nucleus appears as a well-defined circular structure with clear margins. In the two-cell embryo, the nucleus forms directly after the first mitosis and remains until the second mitosis. As the embryo develops, the nuclear status is increasingly harder to assess. Earlier studies used the overarching term “multinucleation” and multinucleated blastomeres (MNB) to describe embryos with more than one visible nucleus. Multinucleation in the four-cell embryo is well-studied, and presence of more than one nucleus is indicative of poor embryo development, increased chromosomal abnormalities and pregnancy loss [8–10]. Again, TLT has allowed for a more complete picture and showed that multinucleation is more common than previously thought [11–13].

Coticchio et al. have summarized the current knowledge on embryo multinucleation at the two-cell stage in a recent review [14]. The impact of multinucleated cells at the two-cell stage on embryo development [15], implantation rate [16] and live birth rate [17, 18] has been shown. Lately, the term nuclear error phenotype (NEP) has been introduced to separate multinucleation into distinct categories as they are likely of different etiology with different impact on subsequent embryo development and viability. These NEP include binucleation, multinucleation, micronucleation, fragmented or split nucleation and a mixture of different NEP in the two cells, here referred to as mixed error. Binucleation refers to two equal sized nuclei in one cell. Failure to align spindles at zygote transition causes binucleated embryos that are diploid, with the genome being distributed in two nuclei [19]. Indirect evidence that some multinucleated cells contain a diploid genome is the fact that mono-nucleus are often larger than the binucleated nuclei [7].The resulting four-cell embryo can therefore be euploid after the second mitosis [20]. Binucleation may also appear after fusion of mononuclear cells, or after replication without division, so called endomitosis, but these irregularities are captured by time lapse and excluded by the study design herein. Here, the term binucleation refers to the presence of two equally sized nuclei in the same cell after normal cell division. Micronucleation is the presence of additional small nuclei in close conjunction to the normal nuclei and can be the outcome of partial genomic DNA replication of gene-poor genomic regions in the short s-phase of fertilization. This can lead to chromosome breakage, segmental loss and overall genome instability. [7] Micro-nucleus can also result as lagging chromosomes are separated from the rest of the chromosomes during anaphase [21]. Nuclear envelope factors gather around the lagging chromosome and create a micronucleus [22]. The micronucleus is prone to collapse, leading to DNA release into the cytoplasm. In the two-cell embryos, they are common and associated with whole chromosome or segmental aneuploidy [23, 24].

**Fig. 1:**
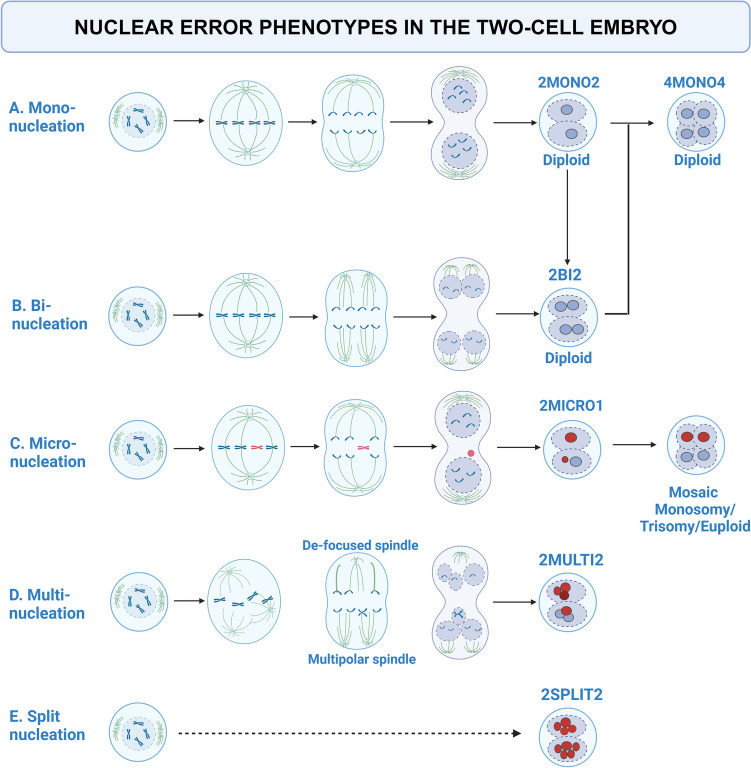
Nuclear error phenotypes in the two-cell embryo with suggested mechanisms of origin At the two-cell stage, the embryo should display one nucleus per cell, referred to as mononucleation (A). The mononucleated embryos are presumably diploid and give rise to a diploid four-cell embryo. Binucleated embryos (B) display two equally sized nuclei in the cell. One mechanism behind binucleated cells at the two-cell stage is failure to align spindles at the zygote transition to cleavage-stage embryo. This results in a diploid two-cell embryos but with two nuclei per affected cell, and after second mitosis the outcome is a diploid four-cell embryo. Division of the nuclei before cell division can also result in binucleation. Micronucleation (C) is the presence of an extra nucleus of smaller size next to the normal nucleus. They contain genetic material and is often associated with whole chromosome or segmental aneuploidy. The resulting embryo is mosaic, containing cells with monosomy, trisomy or euploid genetic content. Multinucleation (D) is the presence of three of more individual nuclei in one cell. They result from de-focused bi-polar spindles, or tri-polar spindles that separates the genetic content into several nuclei. Split nucleation (E) is the pattern of severely fragmented nuclei. The mechanism for split nucleation in embryos is unknown (to be best of our knowledge). Created in BioRender. Adolfsson, E. (2024) https://BioRender.com/k58c062, adapted from Fenech et al. [21] for mechanism of micronucleation, Reichmann et al. [19] and Gomes et al. [20] for mechanisms on binucleation, and Ono et al. [25] for mechanism of multinucleation

Multinucleation, in relation to NEP, refers to three or more equally sized nuclei. This phenotype may result from bi-polar but de-focused spindles or multi-polar spindles at anaphase causing erroneous migration of chromosomes. [25, 26] Finally, we introduce the term split nucleation to describe a pattern of severely fragmented nuclei dispersed in the cytoplasm, and the term mixed error to describe two-cell embryos presenting with different NEP in both cells. See Fig. 1 for an overview of the NEPs used in this study.

The aim of this study was to differentiate multinucleation into NEPs and to investigate the types and frequencies of NEPs at the two-cell stage in fertilized oocytes obtained after ART. Furthermore, the aim was to investigate the impact of NEPs on blastocyst formation rate to provide clinical embryologists with non-invasive methods to improve embryo selection.

## Material and methods

This retrospective cohort study was carried out at the Reproductive Medicine Centre at the University Hospital of Örebro, Sweden. Information on 5080 embryos from 561 consecutive ART cycles in 407 couples from 2018/2019 were collected from the laboratory information system. Unfertilized (*n* = 1464) or abnormally fertilized oocytes (*n* = 423) and oocytes with broken or empty zona pellucida or oocytes degenerating after ICSI (*n* = 262) were then excluded, summing up to *n* = 2149. For the remaining 2931 fertilized oocytes, the first mitotic division was observed. Irregularly divided zygotes not passing through the two-cells stage, i.e. embryos displaying direct cleavage, and two-cell embryos where no nucleus at all could be observed were excluded; embryos where cells fused in order to become two cells or embryos with endomitosis were excluded, *n* = 600. Therefore, the nuclear status of the two-cell embryo was categorized in 2331 zygotes from 504 ART cycles and 392 couples using time lapse images from the EmbryoScope database (Vitrolife, Gothenburg, Sweden).

### Assessment of nuclear status and categorization into nuclear error phenotypes

Nuclear status was examined using time lapse images. For each cell, the nuclear phenotype was noted as mononucleated (presence of one nucleus), binucleated (presence of two equally sized nuclei), micronucleated (presence of micronucleus), multinucleated (presence of > 2 equally sized nuclei) or split nucleation (presence of fragmented nuclei). Each embryo was thereafter categorized as either mononucleated (2MONO2) binucleated (2BI1, 2BI2), micronucleated (2MICRO1, 2MICRO2), multinucleated (2MULTI1, 2MULTI2), split nucleation (2SPLIT1, 2SPLIT2) in one or both cells. Embryos displaying different NEP in the two cells were categorized as mixed error phenotype (2MIXED).

### Ovarian stimulation, oocyte retrieval, fertilization, transfer and/or cryopreservation

Patients underwent controlled ovarian hormone stimulation with either agonist protocol with mid-luteal down regulation or antagonist protocol. Oocyte retrieval was performed 36 h after triggering (human gonadotropin chorionic hormone or agonist trigger) by transvaginal ultrasound guided needle aspiration of available follicles. Sperms were sourced from ejaculation and prepared using density gradient centrifugation. Fertilization method was selected on sperm quality and/or outcome of previous ART cycles. ICSI was done ~ 4 h after oocyte retrieval, and standard IVF was done using overnight co-gamete incubation.

All embryos were cultured in time lapse incubator (EmbryoScope/EmbryoScope + , Vitrolife, Sweden) at + 37 °C, 6% CO_2_ and 5% O_2_ in pre-equilibrated culture media (G-TL™, Vitrolife, Sweden) with oil overlay (OVOIL™, Vitrolife, Sweden). Images were obtained every 15 min in multiple focal plans for at least 44 h post insemination and stored on a local server.

Embryo development, embryo quality and embryo fate (transfer, vitrification or discard) were retrieved from the laboratory information system. (Nuclear status of the two-cell embryo was not part of the embryologist’s assessment of the embryos and did not influence embryo selection.)

### Statistical analysis

For descriptive statistics, continuous variables are presented as median with interquartile range (IQR) and categorical variables as frequencies with percentages. Blastocyst formation rate (BFR) was calculated as the fraction of embryos transferred fresh on day 5 or vitrified on day 5/6 from the pool of normally fertilized embryos, with the exclusion of transferred day 2 or day 3 embryos (*n* = 250).

All categorical variables were analysed using Chi-2 test, followed by post hoc analysis. Association between nuclear status of the two-cell embryo and good quality blastocyst formation rate (BFR) was analysed using contingency table with unadjusted odds ratios (OR) and 95% confidence intervals (CI).

Statistical analysis was done in SPSS (IBM SPSS Statistics for Windows, Version 29.0.2.0 Armonk, USA). *P* values below 0.05 were considered significant.

## Results

### Cohort demographics, cycle characteristics and embryo culture outcomes

Cohort demographics, cycle characteristics and embryo culture outcomes are shown in Table 1. The overall utility rate, i.e. embryos suitable for clinical use after culture, was 24.5% (1247/5080). Fresh transfer was done using 250 cleavage stage embryos (day 2/day 3) and 137 blastocysts (day 5). Cryopreservation by vitrification of good quality blastocysts (Gardner Schoolcraft score > 3BB) was done on day 5 or day 6 of surplus embryos or as part of a freeze-all strategy (*n* = 860). Remaining 1084 embryos were discarded due to arrested development or not fulfilling the criteria for good quality blastocysts.

**Table 1:** Cohort demographics, cycle characteristics and embryo culture outcomes

Female/partner characteristics	Number (n)	Fraction (%)
Patients (n)	392	-
Female age at oocyte retrieval (years)	33 (30–37)	-
Woman’s BMI (kg/m2)	24 (22–28)	-
Female diagnosis (n, %)	100	25.5
Male diagnosis (n, %)	80	20.4
Unexplained (n, %)	198	50.5
Combined (n, %)	12	3.1
Missing (n, %)	2	0.5
Patients with 1 ART cycle in the cycle cohort	303	77.3
Patients with 2 ART cycle in the cycle cohort	69	17.6
Patients with ≥ 3 ART cycle in the cycle cohort	20	5.1
Cycle characteristics		
ART cycles (n)	504	-
Agonist cycles (n, %)	64	12.7
Antagonist cycles (n, %)	440	87.3
Standard IVF (n, %)	258	51.2
ICSI (n, %)	246	48.8
Cycles with only mononucleation	110	21.8
Cycles with NEPs	394	78.2
Cycles with different NEPs in the same cohort	347	68.8
Embryo characteristics		
Total number of oocytes	5080	-
2PN oocytes (n)	2931	57.7
Two-cell embryos categorized (n)	2331	79.5
Irregular cleavage events/no nucleus, discarded (n)	600	21.5
Transferred cleavage stage embryos (n, %)	250	10.7
Clinically useful blastocysts (n, %)	997	42.8
Transferred (fresh, day 5 only)	137	13.7
Vitrified (day 5 or day 6)	860	86.3
Discarded (n, %)	1084	46.5

The median (interquartile range) is given for the woman’s age at oocyte retrieval, BMI, mean oocyte number at oocyte retrieval per cycle. The diagnosis category “female” includes endometriosis (n = 50), anovulation/ovulation disorder (n = 25), ovarian failure (n = 6), tubal factor (n = 16). Clinically useful cleavage stage embryo refers to selected transferred day 2/3 embryos. Clinically useful blastocysts (≥ 3BB Gardner score) were transferred on day 5 or vitrified day 5/6. Discarded refers to arrested and/or poor quality embryos unsuitable for clinical usage.

The good quality BFR was 47.9% (997 good quality blastocysts of 2081 2PN zygotes). Of the 504 cycles, 22% (110/504) presented with only mononucleated cells, 78% (394/504) presented with NE and 69% (347/504) presented with different NEP in the same cohort/cycle. See Table 1.

### Nuclear error phenotype in the two-cell embryo

**Fig. 2:**
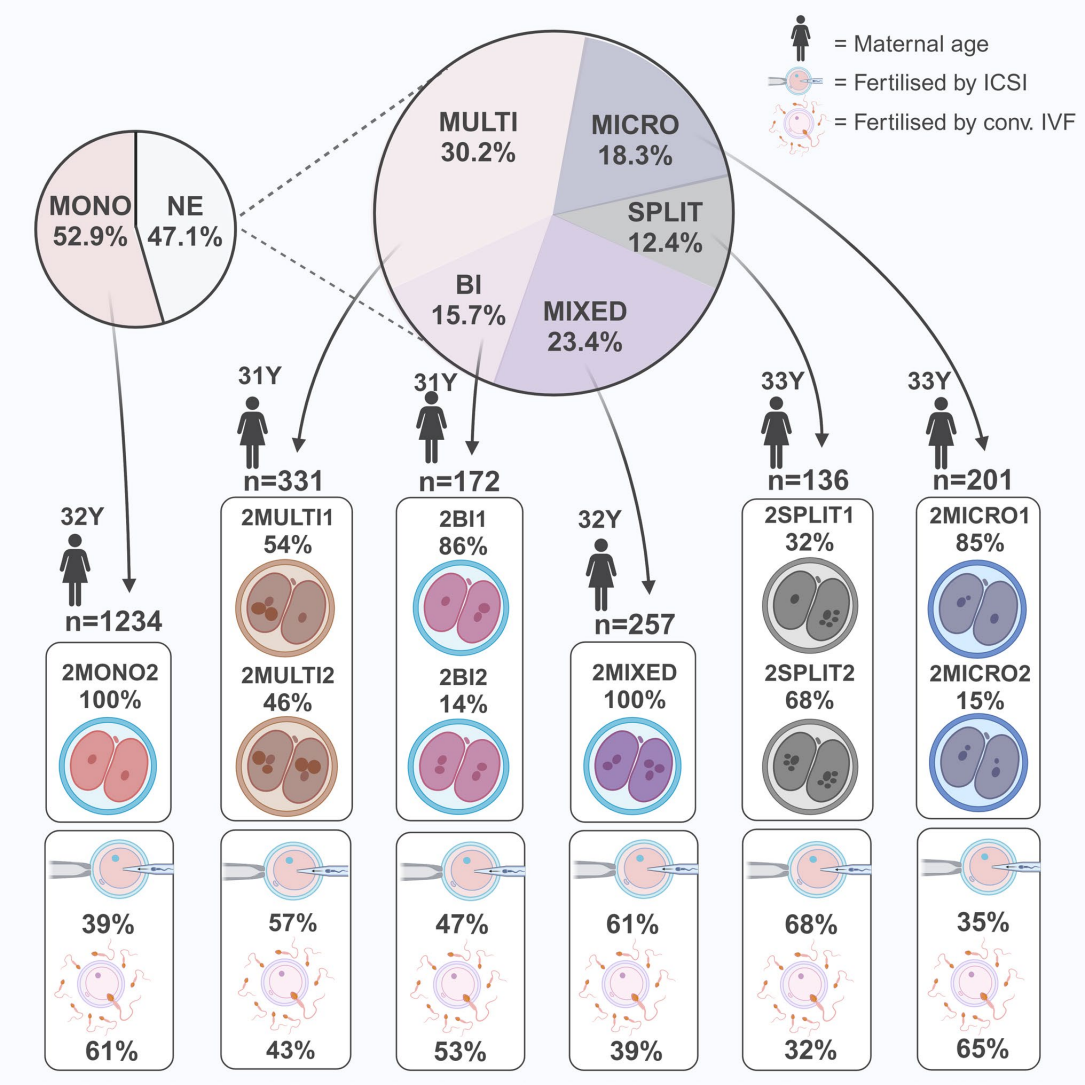
Nuclear error phenotypes in two-cell embryos. The pie chart to the left shows the fraction of mononuclear embryos and embryos with nuclear error. The pie chart to the right shows fraction of each NEP found amongst embryos with nuclear errors. Each category is presented with number of embryos in the group, the median maternal age (no significant differences between the NEPs) and the percentage of embryos within each phenotype displaying the error in one cell or two cells. Percentage of each NEP resulting from fertilization by ICSI or conventional IVF are presented below each phenotype, as well as for mononuclear embryos. Each box sums up to 100%. Created in BioRender. Adolfsson, E. (2024) https://BioRender.com/k23i054

Overall, 52.9% (1234/2331) of the two-cell embryos were mononucleated in both cells, whereas 47.1% (1097/2331) of the two-cell embryos displayed NE. Multinucleation was present in 14.2% of the embryos (*n* = 331), and 54% of these were multinucleated in one cell only. Micronucleation was present in 8.6% of the embryos (*n* = 201), 85.1% of these in one cell. Binucleation was present in 7.4% of the embryos (*n* = 172), and 85.5% of these were binucleated in one cell. Split nucleation was present in 5.8% of the embryos (*n* = 136). and 32.4% were split in one cell. Finally, mixed error was observed in 11% of the embryos (*n* = 257). See Fig. 2.

Split nucleation, multinucleation and mixed error were more common after ICSI, whereas micronucleation was more common after IVF, *X*^2^ (*n* = 2331, 5df) = 100 = *p* < 0.001. See Fig. 2.

Maternal age, maternal BMI, infertility diagnosis or hormone stimulation protocol had no effect on the incidence or type of NE (data not presented).

### Correlation between NE, NEP and formation of good quality blastocysts

**Fig. 3:**
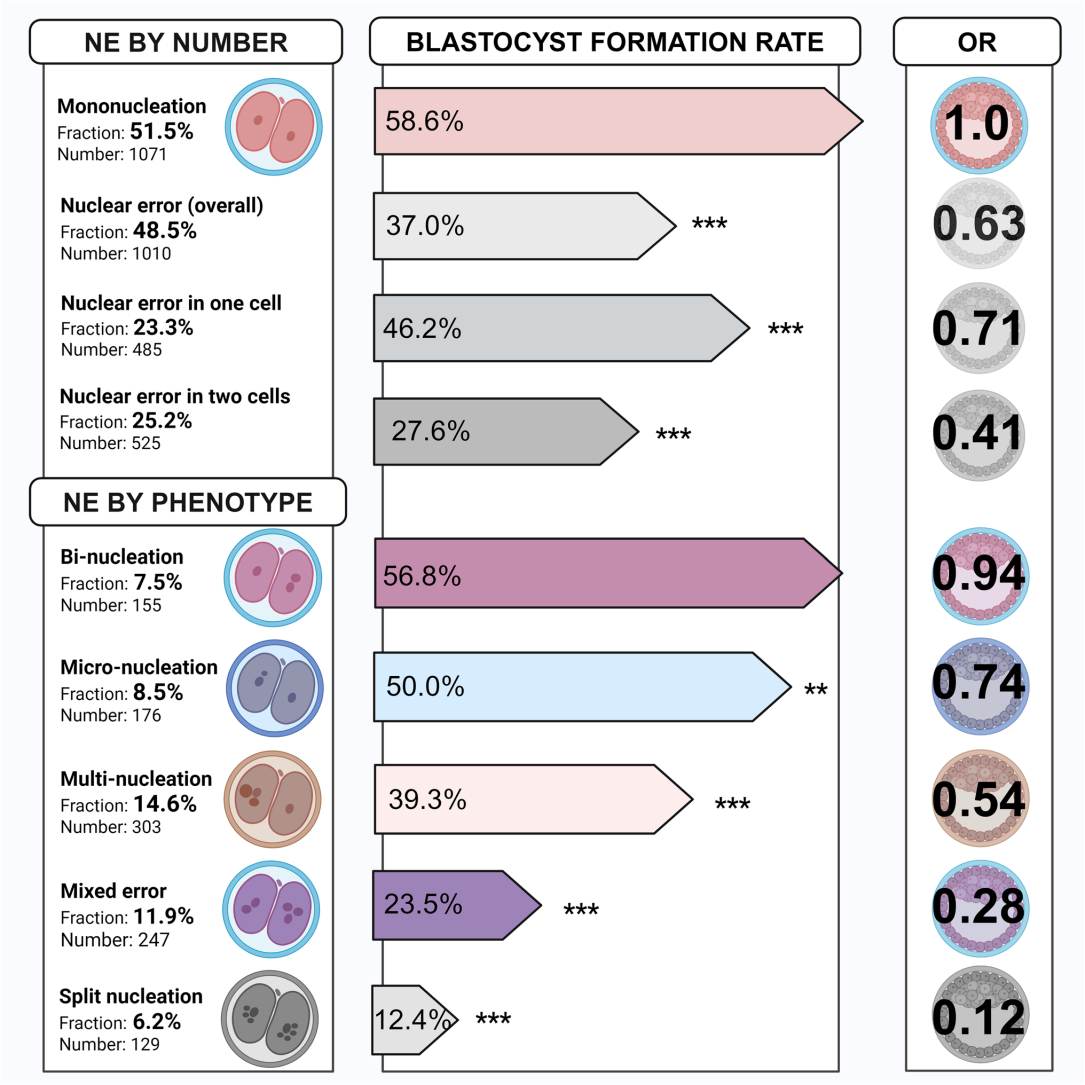
Correlation between nuclear status and blastocyst formation rate Frequencies of nuclear error phenotypes found at the two-cell stage, and the correlation with good quality blastocyst formation rate (in percentage) with odds ratio (OR) relative to mononuclear embryos. Asterisks signify statistical significance: ** = p < 0.05, *** = p < 0.001. Created in BioRender. Adolfsson, E. (2024) BioRender.com/g28w287

Mononucleated embryos had a BFR of 58.6% (628/1071). The presence of NE reduced the BFR to 37% (369/1010) *X*^2^ (*n* = 2081, 1df) = 101 = *p* < 0.001 with an unadjusted OR of 0.63 (0.57–0.69) relative to mononucleated embryos. Embryos with NE in one cell were more likely to develop into blastocysts (224/485, BFR 46.2%) compared to embryos with NE in both cells (145/525, BFR 27.6%), both however, lower compared to normal embryos, unadjusted OR 0.71 (0.61–0.82) and OR 0.41 (0.35–0.48), *p* < 0.001. See Fig. 3.

BFR differed significantly between different NEPs, *X*^2^ (*n* = 1010, 4df) = 92 = *p* < 0.001. Post hoc analysis showed that binucleated and micronucleated two-cell embryos were more likely to become blastocysts (56.8% and 50%, respectively), whereas embryos with multinucleation (39.3%), mixed error (23.5%) or split nucleation (12.4%) were less likely to become blastocysts compared to the other NEPs. See Fig. 3.

Binucleated two-cell embryos did not have a significantly reduced OR relative to mononucleated embryos to develop into a good quality blastocyst. The remaining NEPs did, however. Micronucleated two-cell embryos had a relative risk of 0.74 (0.56–0.98) compared to mononucleated embryos, *p* = 0.02. Multinucleated embryos had a relative risk of 0.54 (0.44–0.67), mixed error a relative risk of 0.28 (0.22–0.37) and split nucleation a relative risk of 0.12 (0.07–0.21), *p* < 0.001 compared to mononucleated embryos. See Fig. 3.

## Discussion

### Nuclear errors and outcome

The overall NE rate in our study was 47.1%. Among the included cycles, 78% had at least one embryo with NE. Using time lapse images, we studied each nucleus, in each cell, in each embryo which enabled us to categorize every two-cell embryo as either binucleated, micronucleated, micronucleated, or carrying split nucleation in one or two cells, or mixed error. NE in one cell was found in 49% and in two cells in 51%. The type of NE varied, with predominantly multinucleation (30.2%), micronucleation (23.4%) and less split nucleation (12.4%). Two-cell embryos with NE in one cell were less able to develop into good quality blastocyst (OR 0.71) and even less able so if they had NE in both cells (OR 0.41) relative to mononucleated embryos. Recent research has shown that the contribution of the daughter cells to the blastocyst after the first mitosis is uneven. Correction mechanisms and formation of blastocysts with excluded cells may compensate for some errors. This may explain why embryos with errors in two cells were less likely to form blastocysts compared to embryos with errors in one cell only. [27] Combining research on NE and observations on complete or partial morula could be of interest to confirm our findings of poorer blastocyst rate from embryos with NE in two cells to investigate if embryos with NE in one cell more often result in a partial morula/blastocyst with excluded cells.

The type of NE also affected the outcome. Binucleated embryos were as likely as mononucleated embryos to become clinically useful blastocysts, whereas the likelihood of an embryo with micronucleation was slightly reduced (OR 0.74), reduced by half for multinucleation (OR 0.54) and the lowest likelihood was found for mixed error (OR 0.28) and split nucleation (OR 0.12) relative to mononucleated embryos.

### Our findings in relation to previous studies

The frequency of nuclear errors is in line with previous studies on cohorts [15, 28, 29] but lower compared to studies on transferred embryos [17]. The majority of previous studies do not separate different phenotypes, and if they do, some phenotypes are combined or omitted. Therefore, comparisons of the NEP frequencies are difficult. Binucleation was reported by Sayed et al. [17] to be the dominant NEP amongst transferred day 2/3 embryos, but we see predominantly multinucleation at two-cell stage, indicating that binucleation may develop into better quality cleavage stage embryos selected for transfer, hence skewing the data on frequencies when using only transferred embryos.

Binucleated embryos are consistently denoted as binucleated in the literature and hence the results easier to compare between our findings and other studies. Talbot et al. found that binucleated two-cell embryos were more likely to develop into good quality blastocysts compared to mononucleated embryos. The binucleated embryos were more likely to implant and give rise to live birth. [18] Sayed et al. [17] noted that binucleated embryos had the same live birth rate as mononuclear embryos, both being higher than micronuclear or multinuclear embryos. Balakier et al. [15] found that binucleated embryos had similar capacity for blastocyst formation as mononucleated embryos, and our findings support this. The mechanisms behind multinucleation support the hypothesis that binucleated cells can carry a diploid genome although split into two nuclei, and the outcome of the second mitosis would then give rise to a normal diploid four cell embryo [20]. We propose that binucleation should not be considered a nuclear error as long as time lapse observations can rule out failed cleavage or fused mononuclear cells (which would result in tetraploid genetic content).

We analysed the developmental potential of two-cell embryos to develop into good quality blastocysts. Our results are in line with Egashira et al. [16] who reported a reduction from 73% BFR to 51% for embryos with NE, but without separation of NEPs. Our findings are contradictory to Aguilar et al. [28], who concluded that NE at the two-cell stage does not affect implantation rate. They did however not separate NEP other than binucleation and multinucleation, and they included micronucleation in the multinucleation cohort.

In our study no apparent differences in maternal age, infertility diagnosis, maternal BMI or stimulation protocol was observed (although the absolute majority of patients were stimulated with antagonist protocol). Fertilization method, however, differed between the observed phenotypes with more split nucleation and mixed error observed after ICSI. It is not possible to identify the underlying cause for this. However, the laboratory procedures before and during ICSI may come with extra stress for the early embryo with more exposure to temperature fluctuations during handling and above all the injection of sperm into the oocyte possibly disrupting the spindle formation or chromosomal alignment on the metaphase plate. Quality does not improve with culture, and more invasive procedures carries extra risks.

### Strengths and limitations

Strengths of the study design include the careful annotation of each cell and the use of NEP categories to capture different types of errors in the embryos which allows for separation of different origins and impacts. The use of the term NE and NEP instead of multinucleation/MNB can hopefully aid other researchers to connect the dots between the genetic background of nuclear errors, the observed pattern in the clinical laboratory and the clinical outcomes for our patients.

We studied an unselected cohort consisting of 2 years consecutive ART cycles in a mid-sized university hospital clinic equipped with time lapse for 10 + years. All embryos were handled with the same procedures/protocols and cultured in the same settings. TLT studies can be limited by the quality and resolution of the images. The multifocal images from the time lapse incubator made it possible to assess every cell, but there is no way to rule out overlapping nuclei or misinterpreted nuclear patterns due to technical issues. Difficult embryos were assessed by multiple researchers, and just a few embryos were excluded as no nucleus at all could be observed. In this study, embryos displaying irregular direct cleavage or reverse cleavage at the two-cell stage were excluded; this ensures that the observed nuclear errors were not a consequence of failed cleavage nor fused cells but resulted from the first mitosis. Such observations can only be done with TLT.

A limitation to this study is the lack of ploidy information on the embryos. Studies combining NEP with genetic screening must be performed in other countries as PGT-A is not allowed in Sweden. Another limitation is that the main purpose was to investigate frequency and types of NEP in fertilized oocytes, and correlation between NEP and BFR. The study was designed to answer the question about presence of NE in the two-cell embryo. The correlation to blastocyst formation can possibly aid with a non-invasive selection tool for embryologists working in clinics with cleavage-stage transfers. Decisions on which embryo to select for transfer should, in our opinion, take into consideration the nuclear status in both the two-cell and the four-cell stage. However, the impact on implantation was not investigated. As the embryo reaches the blastocyst stage, the NE still affects the implantation ability and potential to give rise to viable pregnancy and live birth [12, 17, 30, 31]. Future studies will focus on the correlation of NEPs and live birth rate, where we will keep the categories from this study and combine with transferred embryos with known implantation data and live birth as the primary outcome. Another limitation to the study was the retrospective design using time lapse images in the EmbryoViewer software where embryo fate is not possible to hide from the researcher. It is possible that the researchers were unconsciously influenced although efforts to keep objectivity were taken.

## Conclusion

This study demonstrates a high frequency of NE in the two-cell embryo after ART. The majority of embryos contained one mononucleated cell and one cell with NE. Embryos with NE were less likely to develop into good quality blastocysts. The type of NE influenced the BFR, with worse outcome for split nucleation and mixed error. However, binucleated embryos were as likely to become good quality embryos as mononucleated embryos, and we propose that binucleation can be considered a normality and not a NEP given that time lapse imaging has ensured a normal first mitosis without cell fusion. This provides important information for clinical embryologists, especially those who have to rely on non-invasive methods for embryo selection.

## Data Availability

Data underlying the findings in our study have not been made available. Anonymized data can be shared under compliance with the General Data Protection Regulation (GPDR) regulations and would in most cases require ethical approval. Contact the corresponding author for more information.

## References

[1] Wang X, et al. Conception, early pregnancy loss, and time to clinical pregnancy: a population-based prospective study. Fertil Steril. 2003;79(3):577–84. 10.1016/s0015-0282(02)04694-0. 12620443 10.1016/s0015-0282(02)04694-0

[2] Inge GB, Brinsden PR, Elder KT. Oocyte number per live birth in IVF: were Steptoe and Edwards less wasteful? Hum Reprod. 2005;20(3):588–92. 10.1093/humrep/deh655. 15689347 10.1093/humrep/deh655

[3] Patrizio P, et al. High rate of biological loss in assisted reproduction: it is in the seed, not in the soil. Reprod Biomed Online. 2007;14(1):92–5. 10.1016/s1472-6483(10)60769-9. 17207339 10.1016/s1472-6483(10)60769-9

[4] Meniru GI, Craft IL. Utilization of retrieved oocytes as an index of the efficiency of superovulation strategies for in-vitro fertilization treatment. Hum Reprod. 1997;12(10):2129–32. 10.1093/humrep/12.10.2129. 9402267 10.1093/humrep/12.10.2129

[5] Ivanova AD, Semenova ML. Chromosomal aberrations as a biological phenomenon in human embryonic development. Acta Naturae. 2023;15(3):27–36. 10.32607/actanaturae.25255. 37908766 10.32607/actanaturae.25255 PMC10615189

[6] ESIG/ALPHA, The Vienna consensus: report of an expert meeting on the development of ART laboratory performance indicators. Reprod Biomed Online, 2017. 35(5): 494–510. 10.1016/j.rbmo.2017.06.015 10.1016/j.rbmo.2017.06.015 28784335

[7] Currie CE, et al. The first mitotic division of human embryos is highly error prone. Nat Commun. 2022;13(1):6755. 10.1038/s41467-022-34294-6. 36347869 10.1038/s41467-022-34294-6 PMC9643329

[8] Jackson KV, et al. Multinucleation in normally fertilized embryos is associated with an accelerated ovulation induction response and lower implantation and pregnancy rates in in vitro fertilization-embryo transfer cycles. Fertil Steril. 1998;70(1):60–6. 10.1016/S0015-0282(98)00100-9. 9660422 10.1016/s0015-0282(98)00100-9

[9] Royen EV, et al. Multinucleation in cleavage stage embryos. Hum Reprod. 2003;18(5):1062–9. 10.1093/humrep/deg201. 12721185 10.1093/humrep/deg201

[10] Saldeen P, Sundström P. Nuclear status of four-cell preembryos predicts implantation potential in in vitro fertilization treatment cycles. Fertil Steril. 2005;84(3):584–9. 10.1016/j.fertnstert.2005.03.059. 16169389 10.1016/j.fertnstert.2005.03.059

[11] Desai N, et al. Analysis of embryo morphokinetics, multinucleation and cleavage anomalies using continuous time-lapse monitoring in blastocyst transfer cycles. Reprod Biol Endocrinol. 2014;12:54. 10.1186/1477-7827-12-54. 24951056 10.1186/1477-7827-12-54 PMC4074839

[12] Ergin EG, et al. Frequency of embryo multinucleation detected by time-lapse system and its impact on pregnancy outcome. Fertil Steril. 2014;102(4):1029-1033.e1. 10.1016/j.fertnstert.2014.06.030. 25086787 10.1016/j.fertnstert.2014.06.030

[13] Goodman LR, et al. Does the addition of time-lapse morphokinetics in the selection of embryos for transfer improve pregnancy rates? A randomized controlled trial. Fertil Steril. 2016;105(2):275-85.e10. 10.1016/j.fertnstert.2015.10.013. 26522611 10.1016/j.fertnstert.2015.10.013

[14] Coticchio, G., et al., Embryo multinucleation: detection, possible origins, and implications for treatment. Human Reproduction, 202410.1093/humrep/deae186.10.1093/humrep/deae186 10.1093/humrep/deae186 39173609

[15] Balakier H, et al. Impact of multinucleated blastomeres on embryo developmental competence, morphokinetics, and aneuploidy. Fertil Steril. 2016;106(3):608-614.e2. 10.1016/j.fertnstert.2016.04.041. 27206619 10.1016/j.fertnstert.2016.04.041

[16] Egashira A, et al. Developmental capacity and implantation potential of the embryos with multinucleated blastomeres. J Reprod Dev. 2015;61(6):595–600. 10.1262/jrd.2015-052. 26346255 10.1262/jrd.2015-052 PMC4685227

[17] Sayed S, et al. Nucleation status of Day 2 pre-implantation embryos, acquired by time-lapse imaging during IVF, is associated with live birth. PLoS ONE. 2022;17(9):e0274502. 10.1371/journal.pone.0274502. 36137104 10.1371/journal.pone.0274502 PMC9498959

[18] Talbot AL, et al. Binucleated embryos at the two-cell stage show higher blastocyst formation rates and higher pregnancy and live birth rates compared to non-multinucleated embryos. Hum Reprod Open. 2022;2022(4):hoac049. 10.1093/hropen/hoac049. 36452346 10.1093/hropen/hoac049 PMC9700381

[19] Reichmann J, et al. Dual-spindle formation in zygotes keeps parental genomes apart in early mammalian embryos. Science. 2018;361(6398):189–93. 10.1126/science.aar7462. 30002254 10.1126/science.aar7462

[20] Gomes Paim LM, FitzHarris G. The impact of embryo binucleation depends upon its origin. Reproduction. 2020;160(1):V1–4. 10.1530/rep-20-0188. 32484161 10.1530/REP-20-0188

[21] Fenech M, et al. Molecular mechanisms of micronucleus, nucleoplasmic bridge and nuclear bud formation in mammalian and human cells. Mutagenesis. 2011;26(1):125–32. 10.1093/mutage/geq052. 21164193 10.1093/mutage/geq052

[22] Gauthier BR and V Comaills Nuclear envelope integrity in health and disease: consequences on genome instability and inflammation. Int J Mol Sci. 2021. 22(14).10.3390/ijms22147281 10.3390/ijms22147281 PMC8307504 34298904

[23] Coticchio G, et al. The first mitotic division: a perilous bridge connecting the zygote and the early embryo. Hum Reprod. 2023;38(6):1019–27. 10.1093/humrep/dead067. 37027836 10.1093/humrep/dead067 PMC10233254

[24] Kort DH, et al. Human embryos commonly form abnormal nuclei during development: a mechanism of DNA damage, embryonic aneuploidy, and developmental arrest. Hum Reprod. 2015;31(2):312–23. 10.1093/humrep/dev281. 26621855 10.1093/humrep/dev281

[25] Ono Y, et al. Shape of the first mitotic spindles impacts multinucleation in human embryos. Nat Commun. 2024;15(1):5381. 10.1038/s41467-024-49815-8. 38918406 10.1038/s41467-024-49815-8 PMC11199590

[26] Lazzaro L, Healey M, Osianlis T. Multinucleation in the two-cell stage embryo matters. Fertil Reproduction. 2022;04(0304):167–167. 10.1142/s2661318222740796.

[27] Junyent S, et al. The first two blastomeres contribute unequally to the human embryo. Cell. 2024;187(11):2838-2854.e17. 10.1016/j.cell.2024.04.029. 38744282 10.1016/j.cell.2024.04.029

[28] Aguilar J, et al. Study of nucleation status in the second cell cycle of human embryo and its impact on implantation rate. Fertil Steril. 2016;106(2):291-299.e2. 10.1016/j.fertnstert.2016.03.036. 27059510 10.1016/j.fertnstert.2016.03.036

[29] Hashimoto S, et al. Multinucleation per se is not always sufficient as a marker of abnormality to decide against transferring human embryos. Fertil Steril. 2016;106(1):133-139.e6. 10.1016/j.fertnstert.2016.03.025. 27060728 10.1016/j.fertnstert.2016.03.025

[30] Fauque P, et al. Is the nuclear status of an embryo an independent factor to predict its ability to develop to term? Fertil Steril. 2013;99(5):1299-1304.e3. 10.1016/j.fertnstert.2012.12.028. 23540611 10.1016/j.fertnstert.2012.12.028

[31] Desch L, et al. Embryo multinucleation at the two-cell stage is an independent predictor of intracytoplasmic sperm injection outcomes. Fertil Steril. 2017;107(1):97-103.e4. 10.1016/j.fertnstert.2016.09.022. 28228320 10.1016/j.fertnstert.2016.09.022

